# Microwave-induced *in situ* amorphization: mechanistic basis, formulation principles, and translational perspective

**DOI:** 10.1016/j.ijpx.2026.100696

**Published:** 2026-10-22

**Authors:** Kiran Suwal, Du Hyung Choi, Ji Min Choi, Seong Hoon Jeong, Nam Ah Kim, Ki Hyun Kim

**Affiliations:** a College of Pharmacy, Mokpo National University, Muan, Jeonnam-Gwangju, 58554, Republic of Korea; b College of Pharmacy, Daegu Catholic University, Gyeongsan–si, Gyeongbuk, 38430, Republic of Korea; c College of Pharmacy, Yonsei University, Incheon, 21983, Republic of Korea; d Department of Biomedicine, Health & Life Convergence Sciences, BK21 Four, Biomedical and Healthcare Research Institute, Mokpo National University, Muan, Jeonnam-Gwangju, 58554, Republic of Korea

**Keywords:** Microwave-induced amorphization, *In situ* amorphization, Amorphous solid dispersion, Poorly water-soluble drugs, Physical stability, Solubility enhancement, Formulation design

## Abstract

Although amorphous solid dispersions (ASDs) improve the oral delivery of drugs with poor water-solubility, their broader application is limited by the physical instability of the amorphous state and challenges associated with the downstream processing of metastable materials. Microwave-induced *in situ* amorphization provides an alternative route to ASD formation by maintaining the drug in a crystalline form before microwave treatment and inducing amorphous conversion within the prepared dosage form. This review traces the development of the approach and integrates mechanistic understanding with the formulation and process factors governing its performance. Available evidence indicates that amorphization proceeds primarily through drug dissolution into a transiently mobile carrier phase, rather than direct melting of the crystalline drug. Successful conversion and retention of the amorphous state require coordinated dielectric activation, carrier mobility, and post-amorphization stabilization. The overall performance depends on drug and excipient properties, moisture content, compact structure, and microwave conditions. To facilitate practical implementation, recent formulation strategies have broadened the available activation routes and reduced reliance on external humidity conditioning. Microwave-induced *in situ* amorphization may therefore complement conventional ASD manufacturing, particularly when storage or downstream processing of metastable amorphous systems remains challenging.

## Highlights

• Microwave-responsive excipients enable dielectric activation and carrier mobility.
• Amorphization proceeds mainly by dissolution into a mobile carrier.
• Design must balance activation, mobility, and post-amorphization stabilization.
• Complete conversion does not guarantee acceptable dosage-form performance.
• Translation requires robust activation windows across dosage forms and devices.

## Graphical abstract

**Figure:**
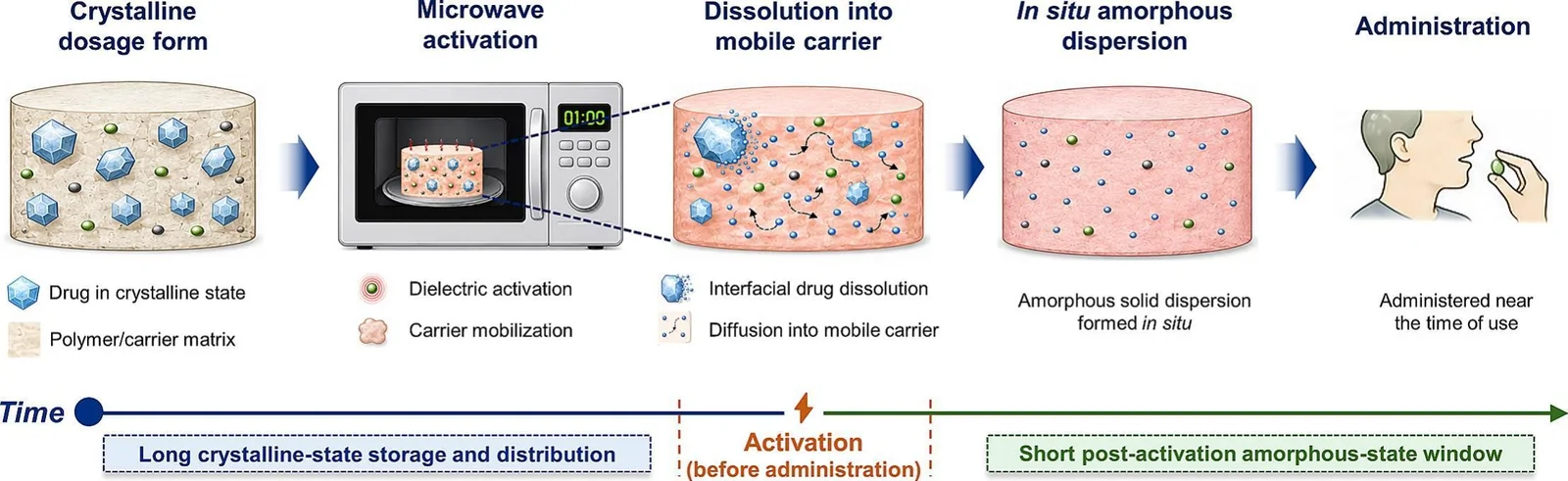

## 1 Introduction

Due to its convenience, non-invasiveness, compatibility with solid dosage forms, and high patient acceptance, oral administration remains one of the most widely used routes for drug therapy (Alqahtani et al., 2021). Despite these advantages, oral absorption is often governed by the extent and rate of drug dissolution in gastrointestinal fluids (Mudie et al., 2010; Onoue, 2024). Poor aqueous solubility therefore remains a persistent barrier in contemporary drug development, especially for Biopharmaceutics Classification System (BCS) Class II and IV compounds, where dissolution-limited absorption often results in variable exposure and insufficient oral bioavailability (Amidon et al., 1995; Bhalani et al., 2022). Among the formulation approaches used to address this problem, amorphous solid dispersion (ASD) technology has become one of the most important enabling approaches, as it harnesses the high-energy amorphous state to increase apparent solubility while relying on polymeric carriers to reduce molecular mobility and suppress recrystallization (Baghel et al., 2016; Moseson et al., 2024).

The biopharmaceutical basis for this improvement lies in the higher Gibbs free energy and disordered molecular arrangement of the amorphous state, which can increase apparent solubility and accelerate dissolution relative to the corresponding crystalline form (Hancock and Parks, 2000; Graeser et al., 2010). However, this same high-energy state also creates the central limitation of amorphous systems: a thermodynamic tendency toward molecular relaxation, phase separation, and recrystallization during processing, storage, or administration (Baghel et al., 2016; Graeser et al., 2010; Yoshioka et al., 1994). Polymeric carriers are therefore incorporated to reduce molecular mobility, promote drug–polymer interactions, and suppress nucleation and crystal growth (Taylor and Zografi, 1997; Yoshioka et al., 1995). In an ideal ASD formulation, the drug is molecularly dispersed within an amorphous polymer matrix, allowing the formulation to retain the dissolution advantage of the amorphous phase while improving its kinetic stability (Bhujbal et al., 2021).

To produce these systems, conventional manufacturing technologies, such as hot-melt extrusion (HME) and spray drying (SD), are widely employed (Bhardwaj et al., 2018; Mendonsa et al., 2020). However, these methods require the product to be generated, processed, and stored in the high-energy amorphous state, imposing a critical dual burden: the formulation must maintain physical stability throughout manufacturing, storage, and distribution, while also withstanding the mechanical and thermal stresses associated with downstream processing operations, such as milling, blending, and compaction (Bhujbal et al., 2021; Sauer et al., 2021). This challenge becomes particularly pronounced at high drug loadings, where reduced drug–polymer miscibility and increased crystallization tendency can constrain the dose range achievable with conventional ASD approaches (Tian et al., 2020). Thus, the major limitation of conventional ASD manufacturing is not merely the difficulty of generating an amorphous dispersion, but the requirement to preserve that metastable state across the entire product life cycle. Triggered amorphization on demand, rather than its continuous maintenance after manufacture, could substantially reduce this fundamental constraint.

An approach based on this principle is *in situ* amorphization, in which the drug is retained in a more stable crystalline form during manufacture and storage, then converted into the amorphous state at or shortly before administration (Qiang et al., 2020). Accordingly, following conversion, the amorphous phase needs to remain stable only for a limited period, rather than throughout the product life cycle. Earlier examples of *in situ* amorphization were reported during dissolution, vaporization, or lipolysis, although the underlying mechanisms were often not fully understood (Priemel et al., 2016). To implement this concept in a controlled manner, an external trigger can be applied to the final dosage form to initiate amorphization at a defined time. Fig. 1 summarizes the conceptual distinction between conventional ASD manufacturing and microwave-induced *in situ* amorphization.

**Fig. 1:**
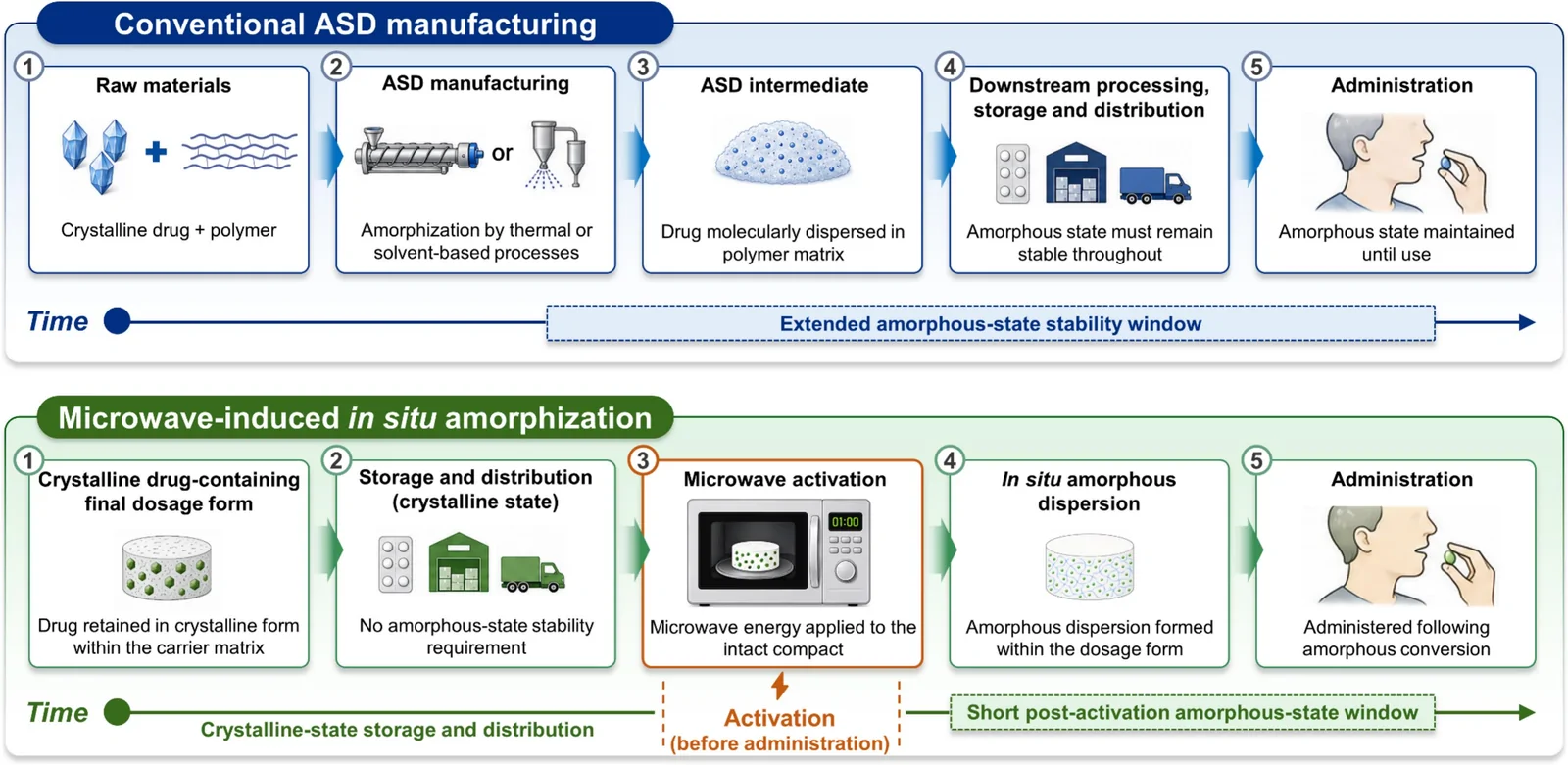
Conceptual comparison between conventional ASD manufacturing and microwave-induced *in situ* amorphization. Conventional ASD manufacturing generates the amorphous dispersion before downstream processing and storage, requiring the metastable amorphous state to be maintained throughout the product life cycle. Microwave-induced *in situ* amorphization instead retains the drug in the crystalline state until activation, shortening the period requiring amorphous-state stability.

Among the potential energy sources for such external triggering, microwave irradiation has attracted particular interest, because it can provide rapid and volumetric heating that acts selectively on dielectrically responsive components within solid dosage forms (Gabriel et al., 1998; Heng et al., 2010). Microwave heating has also been explored to prepare bulk ASD powders through solvent-free, fusion-based approaches (Alshehri et al., 2017; Moneghini et al., 2010; Moneghini et al., 2009; Patil et al., 2013; Shi et al., 2018). However, these approaches usually generate amorphous intermediate powders that still require downstream processing and storage in the metastable amorphous state. In microwave-induced *in situ* amorphization, microwave energy is instead applied to the final dosage form to convert the crystalline drug only when needed. Successful application nevertheless requires careful control of activation conditions, as the extent of amorphization depends on formulation properties, and microwave treatment may alter dosage-form structure, disintegration, and drug release (Doreth et al., 2018; Holm et al., 2022b). Table 1 summarizes the principal differences, potential advantages, and limitations of microwave-induced *in situ* amorphization relative to spray drying and hot-melt extrusion.

**Table 1:** Comparison of microwave-induced *in situ* amorphization with conventional ASD manufacturing technologies.

Attribute	Microwave induced *in situ* amorphization	Spray drying	Hot-melt extrusion
Conversion stage	Within a preformed dosage unit, potentially near administration (Doreth et al., 2017)	Drug and carrier solidify from atomized solution during manufacture (Bhardwaj et al., 2018)	Drug disperses in a softened or molten carrier during manufacture (Bhardwaj et al., 2018; Verreck et al., 2003)
Potential benefit	Reduced need for long-term storage in the amorphous state; fewer subsequent operations on the amorphous material (Doreth et al., 2017)	Flexible solvent-based particle formation; rapid solvent removal (Bhardwaj et al., 2018; Davis et al., 2018)	Typically solvent-free; continuous mixing and processing (Verreck et al., 2003)
Main formulation constraints	Dielectric response, moisture or enabling excipient, carrier mobility, heating uniformity (Doreth et al., 2018; Hempel et al., 2020b; Hempel et al., 2021d; Hempel et al., 2021e; Qiang et al., 2021)	Drug–polymer solubility in the feed; solvent choice, removal, and residual solvent (Bhardwaj et al., 2018)	Thermal and shear exposure; melt viscosity, miscibility, and carrier processability (Verreck et al., 2003)
Dosage-form implications	Activation can change shape, strength, disintegration, and release (Holm et al., 2022b)	Powder usually requires further dosage-form manufacture (Davis et al., 2018)	Extrudate often requires cooling and further dosage-form manufacture (Davis et al., 2018)
Stability and translation	Precursor shelf life plus an activation procedure and a defined interval before dosing require validation (Doreth et al., 2017; Doreth et al., 2018)	Amorphous material must withstand downstream processing and storage (Bhardwaj et al., 2018; Davis et al., 2018)	Amorphous material must withstand downstream processing and storage (Davis et al., 2018; Verreck et al., 2003)

The initial proof-of-concept study showed that microwave irradiation could partially amorphize humidity-conditioned indomethacin–PVP compacts, with the extent of amorphization depending strongly on moisture content and energy input (Doreth et al., 2017). Later studies identified polymer properties, drug loading, particle size, compact structure, temperature, and sorbed water content as important determinants of the degree and rate of amorphization (Doreth et al., 2018; Edinger et al., 2018; Hempel et al., 2020b). Together, these studies indicate that microwave-induced *in situ* amorphization involves heat generation, transient carrier mobility, drug dissolution into the polymeric or molten excipient phase, and stabilization of the amorphous dispersion during cooling (Hempel et al., 2021b; Hempel et al., 2020a; Hempel et al., 2021c; Hempel et al., 2021d; Qiang et al., 2021). In parallel, excipient strategies such as glycerol-containing systems, PEG–PVP composite matrices, crystalline hydrates, and thermolytic salts have been introduced to reduce reliance on external humidity conditioning and improve practical applicability (Hempel et al., 2021d; Hempel et al., 2021e; Holm et al., 2021; Qiang et al., 2024).

Although the experimental literature is still emerging, the studies that have accumulated since these initial demonstrations now allow the determinants of amorphous conversion and post-treatment stabilization to be examined within a common mechanistic framework. This review therefore synthesizes the current literature on microwave-induced *in situ* amorphization around the three interdependent design principles of dielectric activation, carrier mobility, and post-amorphization stabilization. By integrating these mechanistic and formulation-engineering perspectives, it examines how microwave-induced *in situ* amorphization may evolve from a proof-of-concept strategy into a rationally engineered approach for the oral delivery of poorly soluble drugs.

## 2 Development of microwave-induced *in situ* amorphization: From concept to formulation principles

Early work on *in situ* amorphization established the physicochemical basis for inducing amorphous conversion within preformed dosage forms. Prior to the introduction of microwave-assisted processing, Doreth and colleagues demonstrated *in situ* glass solution formation using crystalline drug–polymer compacts without conventional melting or solvent-based manufacturing steps (Doreth et al., 2016). Using naproxen and ibuprofen with Eudragit® E PO, they showed that exposure of compacted formulations to water resulted in the formation of drug–polymer glass solutions. The proposed mechanism involved water penetration into the compact, dissolution of small quantities of crystalline drug, plasticization and swelling of the polymer matrix, and subsequent diffusion of drug molecules into the polymer network. Drug–polymer ionic interactions further facilitated stabilization of the molecularly dispersed amorphous phase. XRPD and DSC analyses showed reduced crystallinity and suggested the formation of single-phase amorphous systems, while FTIR analysis demonstrated ionic interactions between the acidic drugs and the basic polymer. Importantly, this study identified drug-to-polymer ratio, polymer mobility, and drug–polymer interactions as critical formulation parameters governing successful *in situ* amorphization.

Building on this mechanistic foundation, microwave irradiation was subsequently introduced as an external energy source to accelerate *in situ* amorphization within pharmaceutical compacts. In the initial proof-of-concept study, Doreth et al. showed that humidity-conditioned indomethacin–PVP K12 compacts could be partially transformed into a glass solution upon microwave irradiation (Doreth et al., 2017). The extent of amorphization depended strongly on moisture content and microwave energy input, indicating that the process was highly sensitive to both formulation and processing conditions. Spatially heterogeneous conversion and compact deformation also occurred under certain high-energy or high-moisture conditions. This coupled influence of moisture content and microwave energy is illustrated in Fig. 2. Increasing conditioning humidity and microwave energy progressively reduced residual indomethacin crystallinity, with approximately 24% residual crystallinity observed in PVP K12 compacts conditioned at 54% RH and exposed to 90 kJ (Doreth et al., 2017). Importantly, equivalent total energy input delivered at increasing microwave power also produced greater particle fusion, pore formation, and compact deformation, demonstrating that the processing outcome depends not only on total energy input but also on how that energy is delivered. This work established the feasibility of microwave-induced *in situ* amorphization while identifying moisture control, energy input, and dosage-form integrity as issues requiring further systematic investigation.

**Fig. 2:**
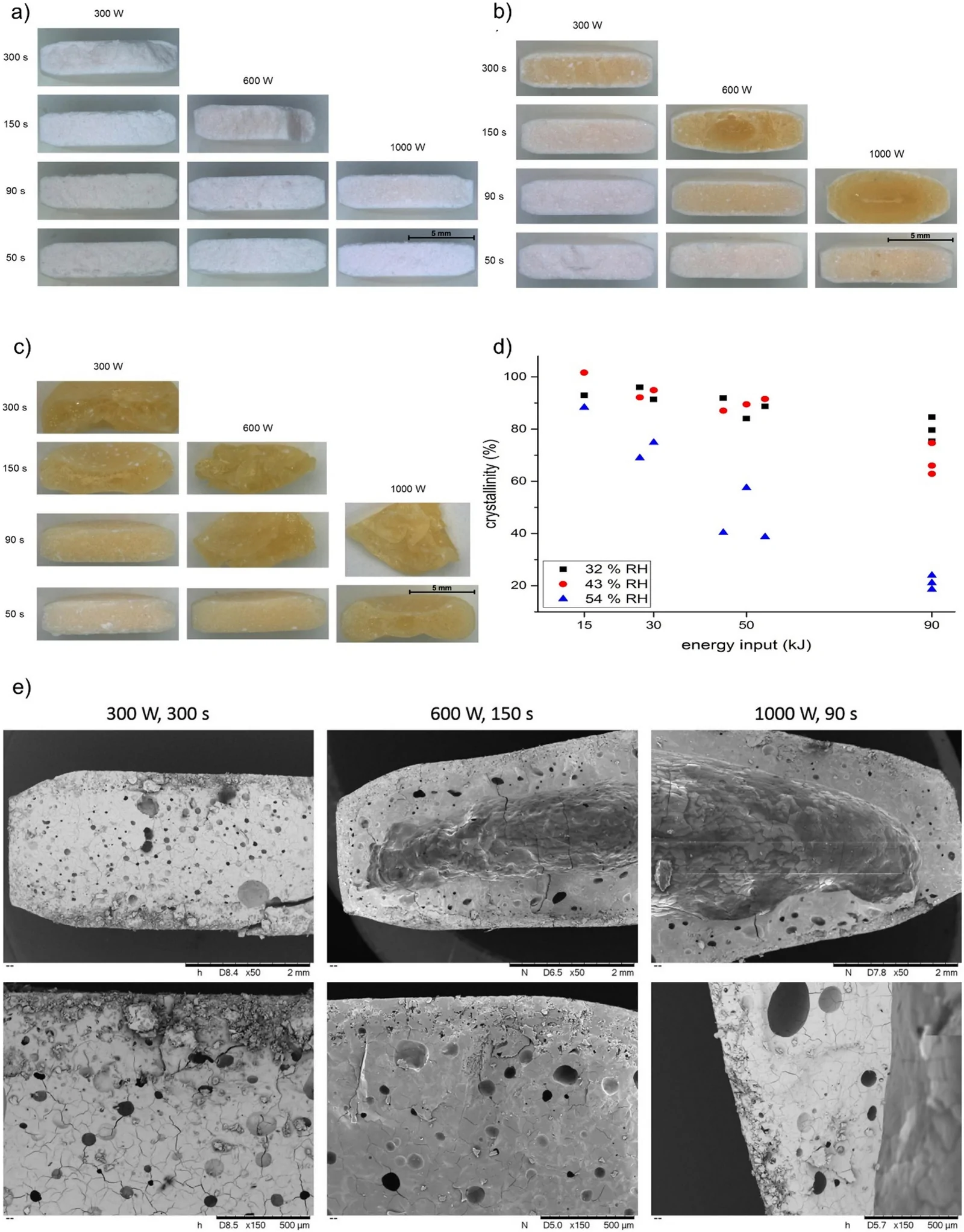
Influence of conditioning humidity and microwave energy input on microwave-induced *in situ* amorphization of indomethacin (IND)–PVP K12 compacts. Cross-sectional optical images of compacts conditioned for at least two weeks at (a) 32%, (b) 43%, and (c) 54% relative humidity (RH), followed by irradiation using the power–time combinations shown. Increasing humidity and energy input were associated with a yellow, glassy appearance. (d) Residual IND crystallinity determined by XRPD as a function of microwave energy input. Compacts conditioned at 54% RH and irradiated at 1000 W for 90 s (90 kJ) exhibited approximately 24% residual crystallinity. (e) SEM images of cross-sections of compacts conditioned at 43% RH and irradiated at an equivalent total energy input of 90 kJ using 300 W for 300 s, 600 W for 150 s, or 1000 W for 90 s, showing particle fusion, pore formation, and compact deformation. Adapted from Doreth et al. (2017), Copyright © 2017 Elsevier B.V., with permission from Elsevier. (For interpretation of the references to colour in this figure legend, the reader is referred to the web version of this article.)

Efforts then shifted toward quantitative assessment and identification of the formulation variables controlling the process. Edinger and co-workers introduced transmission Raman spectroscopy (TRS) combined with partial least squares (PLS) modeling to quantify the degree of amorphization in intact celecoxib–PVP tablets, enabling the non-destructive assessment of microwave-induced conversion (Edinger et al., 2018). The results showed a strong dependence on drug loading, indicating the importance of polymer availability and drug–polymer contact. Studies comparing PVP grades likewise demonstrated that polymer properties, in particular, molecular weight and moisture-dependent mobility, affected the ability of the matrix to support amorphization during microwave irradiation (Doreth et al., 2018). These findings moved the field beyond qualitative feasibility testing and established moisture content, polymer mobility, drug loading, and drug–polymer contact as central determinants of amorphization.

Subsequent work addressed carrier properties, formulation microstructure, and processing conditions. Investigations of polymer function and particle-size effects showed that drug–polymer contact and matrix composition markedly influenced conversion (Hempel et al., 2020b; Qiang et al., 2021), whereas studies on melt-forming carrier systems and comparisons with convective heating further identified carrier viscosity and thermal history as additional determinants (Hempel et al., 2021b; Hempel et al., 2020a). Parallel work broadened polymer screening and extended evaluation to the mechanical and drug-release performance of microwave-treated tablets (Zhang et al., 2021). The sensitivity of humidity-conditioned systems to the conditioning procedure and to water loss during irradiation (Doreth et al., 2017) prompted the development of internally engineered formulations based on alternative carriers, plasticizers, water-releasing excipients, and water-generating strategies (Hempel et al., 2021d; Hempel et al., 2021e; Holm et al., 2021; Qiang et al., 2023; Qiang et al., 2024). Building on hydrate-based internal activation, subsequent studies extended the concept to highly loaded supersaturated ASDs and then to multi-particulate dosage forms, shifting attention from amorphous conversion alone to the coupled control of dosage-form integrity, disintegration, and dissolution (Holm et al., 2022a; Holm et al., 2022b).

Table 2 summarizes the formulation compositions, initial water contents, microwave-processing conditions, quantitative amorphization outcomes, and available post-treatment stability and product-quality observations reported across the published studies. The reported amorphization outcomes should not be interpreted as directly comparable measures of performance, as the studies used different analytical methods and reporting criteria.

**Table 2:** Experimental conditions and amorphization outcomes reported in microwave-induced *in situ* amorphization studies.

Drug	Carrier / additional excipients	Drug loading (%, *w*/w)	Initial water content (% w/w)	Microwave power (W)	Processing time (min)	Amorphization outcome	Post-treatment stability / quality observation	Reference
Indomethacin	PVP K12	33.3	7.0–9.8	300; 600; 1000	0.83–5	Up to 80%	No microwave-induced drug degradation detected	(Doreth et al., 2017)
Indomethacin	PVP K12, K17, K25	33.3	9.9–12.3	300	5, 10	Up to 58%	Delayed initial release due to poor disintegration	(Doreth et al., 2018)
Celecoxib	PVP K25	10–50	12.4–20.8	1000	1–10	Up to 90%	NR	(Edinger et al., 2018)
Celecoxib	PVP K12	30	22	1000	10	Up to 100%	NR	(Hempel et al., 2020a)†
Celecoxib	PVP K12	30	22	1000	1–10	Up to 100%	NR	(Hempel et al., 2020b)
Celecoxib	PEG 3000, 4000	30	NA	1000	2–10	Up to 100%	NR	(Hempel et al., 2021b)
Indomethacin	Soluplus®; Glycerol	20.0–50.1	NA	1000	5–20	Up to 100%	20% IND remained amorphous for 2 weeks; 30% IND recrystallized	(Hempel et al., 2021e)
Indomethacin	Soluplus®; HPC HF; HPMC K4M; HPMCAS; Eudragit® E PO; PEO N10	30	NA	1000	14–35	Up to 100%	Improved drug release	(Zhang et al., 2021)
Celecoxib	PVP K12, K17	30	19.4–20.5	1000	≤10	Up to 100%	NR	(Hempel et al., 2021c)
Celecoxib	PVP K17; PEG 2000, 3000, 4000	30–50	0.9–4.7	1000	2–10	Up to 100%	Recrystallized on cooling in PEG-only systems; PVP improved amorphous retention	(Hempel et al., 2021d)
Celecoxib	PVP K12; NaH_2_PO_4_	16.6–29.9	9.7–11.1	900	2–10	Up to 100%	NR	(Holm et al., 2021)
Indomethacin	PVP K30; PVAc; PVP/VA 64, I-335, I-535, I-735	20	0.6–16.6	1000	≤20	Up to 100%	No significant drug loss after conditioning/microwave treatment	(Qiang et al., 2021)
Celecoxib	PVP K12; PVP/VA 64; PVAc; NaH_2_PO_4_	30–90	NA	1000	10	Up to 100%	Fusion-related poor disintegration delayed drug release	(Holm et al., 2022a)
Celecoxib	PVP/VA 64; NaH_2_PO_4_; MCC; Mannitol	18.8	NA	800; 1000	10	Up to 100%	Fusion impaired disintegration and dissolution	(Holm et al., 2022b)
Indomethacin	PVP/VA I-535; non-ionic surfactants⁎	20	3.0–4.7	1000	20	Up to 100%	Fast, complete dissolution with Kolliphor RH40	(Qiang et al., 2023)
Indomethacin	PVP/VA I-535; NH_4_HCO_3_	20–50	NA	1000	15, 20	Up to 100%	Improved chemical stability *vs* moisture-based systems; physical stability: NR	(Qiang et al., 2024)
Indomethacin	PVP/VA 64	20	8.4–13.5	900	20	Up to 100%	Improved dissolution despite marked tablet deformation	(Zhang et al., 2026)

NA: not available; NR: post-treatment stability or relevant product-quality observations were not reported or quantitatively evaluated.

⁎ Kollisolv® P124, Kolliphor® RH 40, TPGS, and Tween 60, 65, 80, and 85.

† Microwave data in Hempel et al. (2020a) were originally reported in Hempel et al. (2020b).

Overall, microwave-induced *in situ* amorphization has progressed from a proof-of-concept technique to a formulation-dependent process in which material selection, moisture management, energy input, and dosage-form design are interdependent requirements. This progression explains why irradiation conditions in isolation cannot predict the outcome. Successful conversion first requires the formulation to translate absorbed microwave energy into a transient physicochemical state that permits amorphization within the intact compact. The resulting amorphous phase must then be retained after treatment. The molecular and material basis of these linked requirements forms the mechanistic framework discussed in the following section.

## 3 Mechanistic basis of microwave-induced *in situ* amorphization

### 3.1 Amorphization as dissolution into a mobile carrier phase

The available evidence is most consistent with microwave-induced amorphization occurring through drug dissolution into a transiently mobile carrier phase, rather than through direct melting of the crystalline drug (Hempel et al., 2020a; Hempel et al., 2020b; Moseson et al., 2019). This distinction shifts the focus from the melting behavior of the drug itself to the physicochemical properties of the surrounding matrix, in particular the carrier mobility, viscosity, temperature, water content, and drug–carrier contact. Under microwave irradiation, the carrier must first become sufficiently softened or plasticized to accommodate drug molecules. Once this mobile state is reached, a crystalline drug can progressively dissolve into the carrier and become molecularly dispersed, thereby forming an amorphous solid dispersion *in situ*. Accordingly, the rate and extent of amorphization can be interpreted conceptually within a Noyes–Whitney-type framework, in which drug transfer into the carrier is influenced by interfacial area, diffusion distance, matrix viscosity, and temperature, rather than by microwave exposure alone (Noyes and Whitney, 1897). This framework is intended as a qualitative description of interfacial mass transfer, rather than a direct quantitative application of the classical aqueous dissolution equation. The maximum achievable amorphous fraction is also formulation-dependent and may be limited by drug loading, drug–carrier miscibility, and the amount of carrier available for molecular dispersion (Edinger et al., 2018; Holm et al., 2022a). At high drug loading, incomplete conversion may therefore reflect both slower dissolution kinetics and insufficient capacity of the mobile carrier phase to accommodate the drug. Fig. 3 shows a schematic of this sequence.

**Fig. 3:**
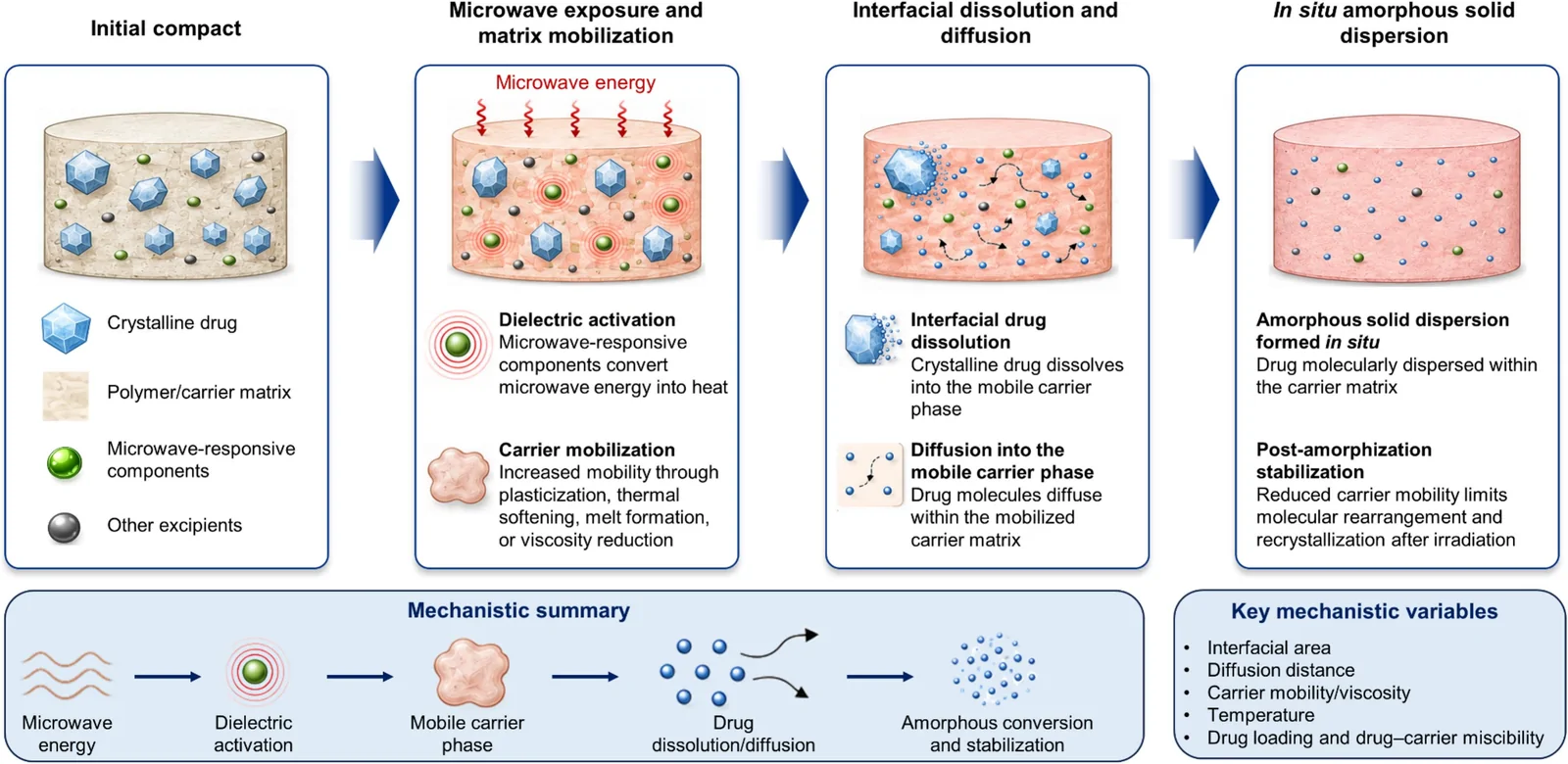
Dissolution-controlled mechanism of microwave-induced *in situ* amorphization. Microwave-responsive components convert microwave energy into heat, mobilizing the carrier phase so that crystalline drug dissolves at the drug–carrier interface and diffuses within the matrix. Reduced carrier mobility after irradiation limits recrystallization. Key variables governing the rate and extent of conversion are listed.

Evidence for this dissolution-controlled interpretation is provided by the particle-size study of Hempel et al. (2020b), in which reducing drug and polymer particle size increased drug–polymer interfacial contact and promoted more extensive amorphization (Fig. 4). After 600 s of microwave irradiation at 1000 W, complete celecoxib amorphization was achieved when both celecoxib and PVP were present as small particles, whereas approximately 68% amorphization was obtained with small celecoxib/large PVP particles and only approximately 25% when large celecoxib particles were used. Notably, compacts containing larger drug and polymer particles showed less amorphization despite reaching higher temperatures after longer irradiation (Fig. 4d), indicating that maximum temperature alone did not fully explain conversion. Rather, the strong influence of drug particle size is consistent with an interfacial dissolution and mass-transfer process, in which increased drug surface area facilitates dissolution into the mobile polymer phase and reduces the effective diffusion distance required for incorporation into the carrier matrix. This behavior is consistent with the Noyes–Whitney framework, whereby increased interfacial area can enhance dissolution, and is difficult to reconcile with a mechanism governed solely by direct melting of the crystalline drug, for which particle-size reduction would not be expected to exert the same dominant effect on conversion (Moseson et al., 2019; Noyes and Whitney, 1897). Thus, the particle-size dependence shown in Fig. 4 provides experimental support for dissolution of crystalline drug into a transiently mobile carrier phase and establishes a direct mechanistic link between formulation microstructure, drug–polymer contact, and amorphous conversion.

**Fig. 4:**
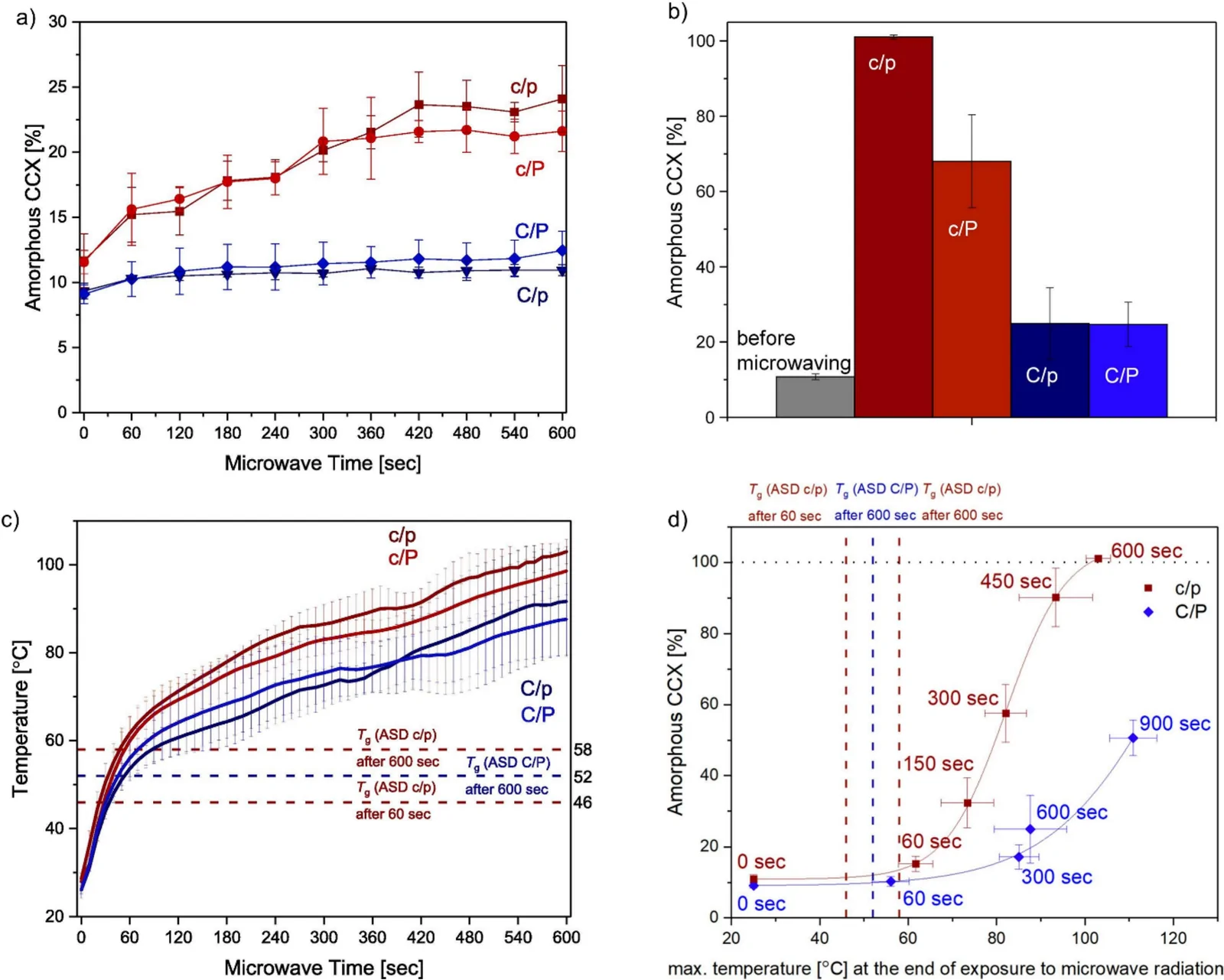
Influence of drug and polymer particle size and compact temperature on microwave-induced *in situ* amorphization of celecoxib (CCX) in PVP. (a) Degree of CCX amorphization, quantified by transmission Raman spectroscopy, during repeated 60-s irradiation intervals up to a cumulative exposure time of 600 s. (b) Degree of amorphization before and after continuous irradiation at 1000 W for 600 s. Complete amorphization was achieved for c/p, compared with approximately 68% for c/P and 25% for C/p and C/P. (c) Core temperature profiles during continuous irradiation for 600 s, together with the indicated glass-transition temperatures (T_g_). (d) Relationship between the degree of amorphization and the maximum core temperature for c/p and C/P at different irradiation times (up to 900 s). Lowercase c and p denote small CCX and PVP particles (<71 μm), respectively, whereas uppercase C and P denote the corresponding large particle fractions (>71 μm). Adapted from Hempel et al. (2020b), Copyright © 2020 Elsevier B.V., with permission from Elsevier.

Comparisons between microwave irradiation and conventional convective heating further support this interpretation. Microwave exposure alone was insufficient to induce amorphization unless the compact reached the temperature and mobility conditions required for drug dissolution into the carrier (Hempel et al., 2020a). Substantial amorphization below the drug melting point is consistent with dissolution into the carrier, rather than direct drug melting. A separate study used the appearance of completely melted pure indomethacin as a visual reference to determine the heating endpoint of formulated tablets, thereby leaving open the possibility that direct drug melting contributed under those conditions (Zhang et al., 2021). In systems processed below the drug melting point, microwave irradiation therefore primarily functions as a means of generating the physicochemical state required for dissolution-controlled amorphization, rather than as an independent amorphizing force. Available comparisons with convective heating do not provide evidence for a distinct non-thermal microwave effect in these systems. Its potential advantage therefore lies in rapid volumetric energy deposition and preferential heating of microwave-responsive components within the intact dosage form. This distinction is particularly relevant when amorphization is intended to occur in a final or near-final compact, rather than in a bulk material before dosage-form preparation. The relevant question is whether the formulation can convert absorbed microwave energy into a carrier environment with sufficient molecular mobility to accommodate and disperse the drug.

### 3.2 Dielectric activation: Microwave-responsive components as energy transducers

Microwave-induced *in situ* amorphization requires effective coupling between the applied electromagnetic field and formulation components that can convert microwave energy into heat. These microwave-responsive components therefore act as energy transducers within the dosage form. In the reported systems, microwave treatment has typically been performed at 2.45 GHz, at which frequency heating can arise through mechanisms like dipolar polarization and ionic conduction (Gabriel et al., 1998). In systems containing polar components, such as water or glycerol, dipolar polarization is likely to be the dominant contribution, although when mobile ionic species are present, ionic conduction may also become relevant. Dielectric activation is mechanistically distinct from the subsequent plasticization and diffusion processes: it governs heat generation, whereas carrier mobility determines whether the resulting thermal input can promote drug dissolution and amorphous conversion. Successful amorphization therefore depends not just on the presence of microwave-responsive components, but also on whether they generate a thermal profile suitable for conversion within the compact.

Sorbed water provided the earliest and most direct route to dielectric activation in microwave-induced *in situ* amorphization. In humidity-conditioned indomethacin–PVP compacts, absorbed water enabled microwave-induced amorphization, consistent with its role as a polar, microwave-responsive component (Doreth et al., 2017). The strong dependence of amorphization on moisture content indicates that water acted as an active mediator of microwave energy absorption, rather than a passive formulation variable. However, this approach introduced practical limitations, including variability in water distribution, moisture loss during irradiation, and sensitivity to the humidity-conditioning procedure. These limitations motivated strategies in which dielectric activation is provided by excipients incorporated into the formulation, rather than by externally sorbed water (Holm et al., 2021).

One such strategy is to incorporate water into the solid-state structure of an excipient. Crystalline hydrates can release crystal water during microwave exposure, thereby supplying an internal source of dielectric activation without prior humidity conditioning (Holm et al., 2021). Thermolytic salts represent a related, though chemically distinct, strategy. Rather than releasing pre-existing crystal water, they generate water and other volatile decomposition products upon heating, creating an internally generated microwave-responsive phase during treatment (Qiang et al., 2024). Both approaches replace externally sorbed water with microwave-responsive species that are internally available or generated during irradiation. In NH_4_HCO_3_-containing systems, microwave irradiation can also induce formulation- and time-dependent formation of ammonium indomethacin salt (Qiang et al., 2024). Amorphization and salt formation should therefore be distinguished when interpreting dissolution behavior. In that study, the formulation containing 50% (*w*/w) indomethacin was polymer-free and should therefore not be interpreted as a polymeric ASD at that drug loading.

Non-aqueous components provide additional routes to dielectric activation. In PEG-based melt-forming systems, microwave treatment promoted melt formation and increased carrier mobility, reducing reliance on water-mediated activation (Hempel et al., 2021b; Hempel et al., 2021d). Polar liquid excipients, such as glycerol, provide another route, because their polarity supports microwave coupling while reducing dependence on external humidity conditioning (Hempel et al., 2021e). Non-ionic surfactants have also been evaluated as microwave-responsive excipients with combined dielectric-heating and plasticizing functions (Qiang et al., 2023). Fig. 5 summarizes these representative activation routes.

**Fig. 5:**
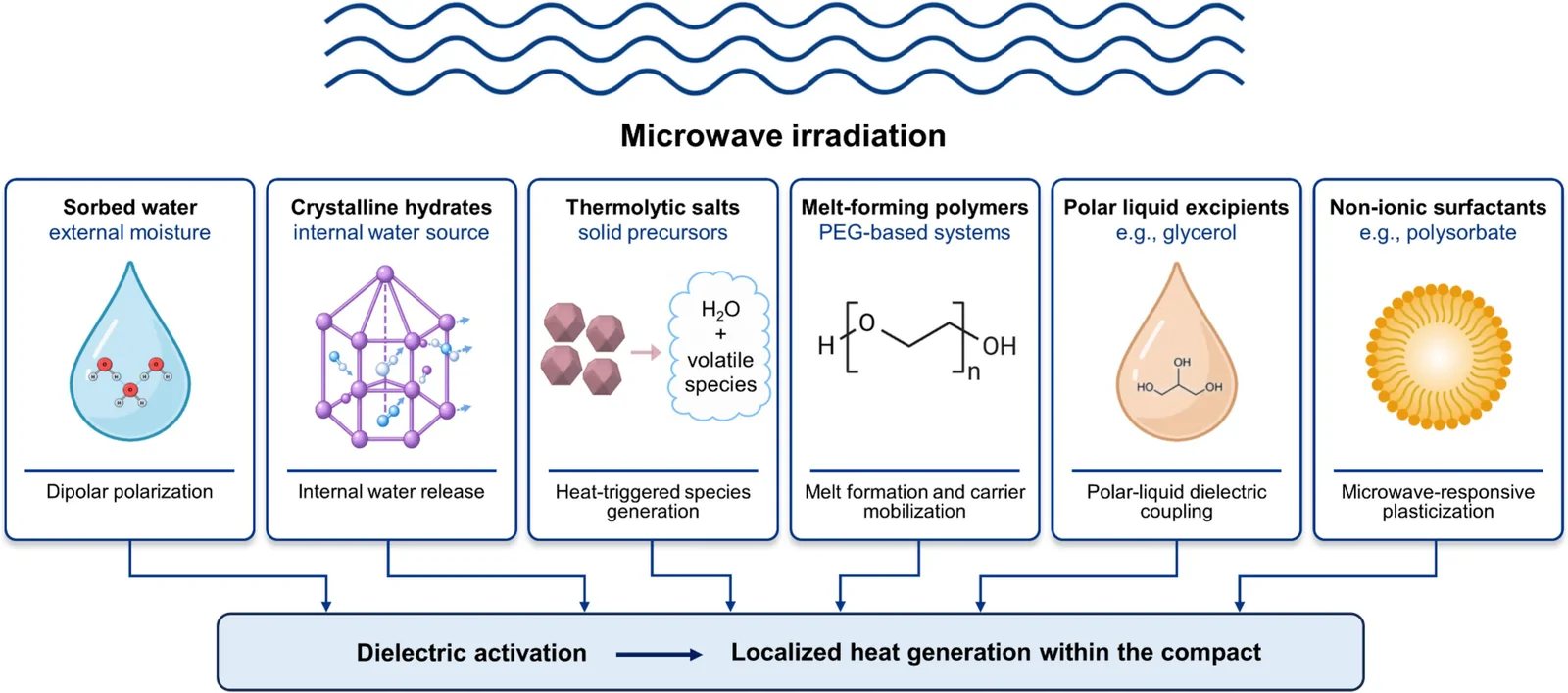
Representative routes to dielectric activation under microwave irradiation. Sorbed water, crystalline hydrates, thermolytic salts, melt-forming polymers, polar liquid excipients, and non-ionic surfactants contribute through distinct dielectric and thermally mediated mechanisms, ultimately converging on localized heat generation within the compact.

Dielectric activation is therefore a formulation-dependent process, rather than a generic consequence of microwave exposure. The central design questions are which components absorb microwave energy, where heat is generated, and whether heating occurs in the appropriate region and for a sufficient period to support the mobile carrier state required for drug dissolution. Microwave-responsive components provide the energetic trigger for *in situ* amorphization, whereas the resulting carrier softening, plasticization, viscosity reduction, and enhanced drug diffusion constitute the subsequent mobility-controlled stage.

### 3.3 Carrier plasticization and mobility: From energy absorption to drug diffusion

Dielectric activation alone does not ensure amorphous conversion (Hempel et al., 2021c). The absorbed microwave energy must generate a carrier state with sufficient molecular mobility to allow drug dissolution and diffusion. In microwave-induced *in situ* amorphization, this mobile carrier state may arise through several related mechanisms, including water-induced plasticization and thermal softening of polymeric carriers, melt formation in low-melting carrier systems, and temperature-dependent viscosity reduction in molten or liquid-containing matrices (Hempel et al., 2021b; Hempel et al., 2021c; Hempel et al., 2021d; Hempel et al., 2021e). These processes reduce transport resistance within the matrix, allowing drug molecules to leave the crystalline phase and become incorporated into the carrier. Carrier mobility represents the mechanistic link between microwave energy absorption and dissolution-controlled amorphization, as illustrated by the transition from dielectric activation to drug dissolution and diffusion in Fig. 3.

Beyond its role in dielectric activation, water is particularly important because it also acts as a plasticizer. Water uptake can lower the glass-transition temperature of amorphous polymeric materials and increase molecular mobility (Hancock and Zografi, 1994). In PVP-based systems, absorbed water can plasticize the polymeric matrix, thereby facilitating drug dissolution into the carrier during microwave exposure (Doreth et al., 2017). This dual role helps explain the close relationship between moisture content and the extent of amorphization in these systems. However, excessive water can lead to compact deformation or heterogeneous conversion. The formulation target is therefore not to maximize water content, but to achieve sufficient mobility without compromising the physical integrity of the dosage form.

Polymer properties strongly influence how effectively absorbed microwave energy is translated into matrix mobility and, consequently, drug amorphization. In particular, polymer molecular weight affects chain mobility, moisture-dependent plasticization, viscosity, and matrix softening behavior. This relationship is illustrated in Fig. 6, where indomethacin formulations containing different PVP grades were compared under similar humidity-conditioning and microwave-treatment conditions (Doreth et al., 2018). PVP K12 compacts exhibited pronounced structural fusion and progressively greater amorphization with increasing energy input, whereas formulations containing the higher-molecular-weight PVP K17 and K25 largely retained their particulate structure, with indomethacin remaining essentially crystalline. In the PVP K12 formulation, residual indomethacin crystallinity decreased from approximately 69% after 90 kJ to approximately 42% after 180 kJ, corresponding to approximately 31% and 58% amorphization, respectively (Doreth et al., 2018). These findings indicate that polymer molecular weight influences microwave-induced amorphization primarily through its effects on moisture uptake, plasticization, viscosity, and molecular mobility rather than through glass-transition temperature alone. A carrier with insufficient mobility may restrict drug diffusion even when microwave energy is efficiently absorbed. Conversely, excessive matrix softening or fusion may compromise compact geometry and subsequent disintegration, as observed in early humidity-conditioned compacts and later multiparticulate systems (Doreth et al., 2017; Holm et al., 2022b). Polymer selection should therefore balance the ability to develop sufficient transient molecular mobility during microwave irradiation with the capacity to stabilize the resulting amorphous drug while maintaining acceptable dosage-form structure and performance.

**Fig. 6:**
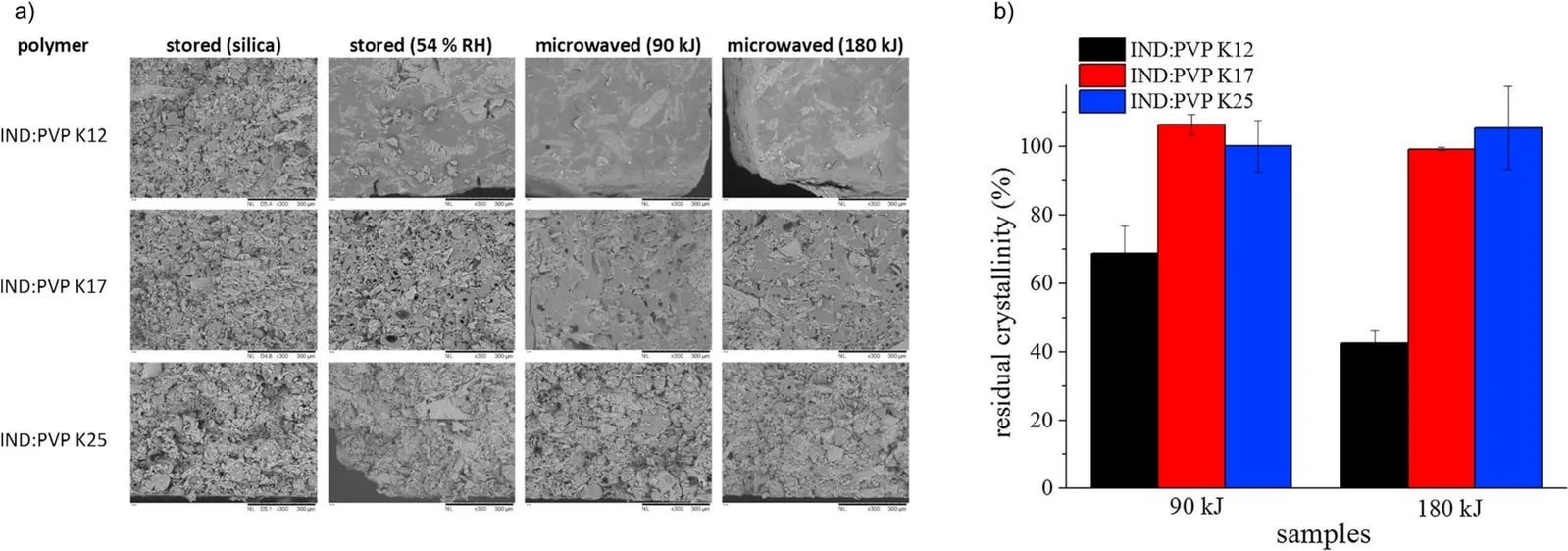
Effect of PVP molecular weight on microwave-assisted *in situ* amorphization of indomethacin (IND). (a) SEM cross-sections of IND–PVP K12, K17, and K25 compacts stored over silica gel or conditioned at 54% RH, together with 54% RH-conditioned compacts irradiated at 300 W for 5 or 10 min (90 or 180 kJ, respectively). Particle fusion was more pronounced in PVP K12 compacts than in K17 and K25 compacts. (b) Residual IND crystallinity determined by XRPD after irradiation. PVP K12 compacts showed approximately 69% and 42% residual crystallinity at 90 and 180 kJ, corresponding to approximately 31% and 58% amorphization, respectively. IND remained essentially crystalline in PVP K17 and K25 compacts. Adapted from Doreth et al. (2018), Copyright © 2017 Elsevier B.V., with permission from Elsevier.

Low-melting and liquid excipients provide additional routes to carrier mobility. In PEG-based systems, the rate of microwave-induced amorphization was strongly influenced by melt formation and temperature-dependent viscosity (Hempel et al., 2021b; Hempel et al., 2021d). Efficient amorphization therefore requires a carrier phase that becomes sufficiently fluid under irradiation to support drug dissolution and diffusion while maintaining an appropriate balance among temperature, viscosity, and drug–carrier miscibility. Glycerol-containing systems extend this principle to polar liquid excipients, which can support matrix plasticization and drug solubilization, while also contributing to microwave responsiveness (Hempel et al., 2021e). These systems show that carrier mobility can be modulated either by plasticizing a polymeric matrix or by incorporating low-viscosity or melt-forming components.

The available evidence supports a mobility-controlled view of microwave-induced *in situ* amorphization. Dielectric activation provides the energy input, but amorphous conversion proceeds only when the carrier reaches a physical state that permits drug dissolution and molecular transport. Similar microwave exposure conditions can produce different amorphization outcomes, depending on the carrier composition, moisture content, polymer grade, and excipient viscosity. The formulation challenge is to generate sufficient carrier mobility during irradiation while preserving compact shape and minimizing spatial heterogeneity in conversion. Once this mobile state has enabled drug dissolution, the resulting amorphous phase must be sufficiently immobilized to suppress phase separation and recrystallization.

### 3.4 Post-amorphization stabilization and recrystallization control

The dissolution-controlled amorphization described above is necessary, but not sufficient, for a pharmaceutically useful dosage form. After crystalline drug has dissolved into a mobile carrier phase, the resulting amorphous phase must be sufficiently stabilized after irradiation. This requirement is mechanistically distinct from dielectric activation and carrier plasticization. Dielectric activation supplies heat, and carrier mobility enables drug dissolution and diffusion; post-amorphization stabilization determines whether the molecularly dispersed drug remains amorphous or undergoes phase separation and recrystallization after treatment.

The central challenge arises because molecular mobility must serve two opposing functions at different stages of the process. During irradiation, increased mobility allows the carrier phase to accommodate drug molecules and promotes amorphous conversion. After irradiation, the same mobility can become detrimental if it permits molecular rearrangement, drug-rich domain formation, nucleation, or crystal growth. Successful formulation design therefore requires a transient increase in mobility during microwave exposure, followed by sufficient immobilization of the drug within the carrier matrix after irradiation (Hempel et al., 2021d; Lalge et al., 2023; Lehmkemper et al., 2017b).

Several formulation variables control this transition from mobility to immobilization. During irradiation, the carrier must provide sufficient mobility to support drug incorporation, but once microwave exposure ends, it must regain sufficient rigidity to limit molecular rearrangement (Hempel et al., 2021d; Lehmkemper et al., 2018; Lehmkemper et al., 2017b). Residual water or low-molecular-weight components may facilitate amorphization during irradiation, yet their persistence after treatment can maintain molecular mobility and thereby increase the risk of recrystallization (Lehmkemper et al., 2018; Lehmkemper et al., 2017a). Poor drug–carrier miscibility or weak intermolecular interactions may also allow phase separation, even when initial amorphization is achieved (Lalge et al., 2023; Lehmkemper et al., 2017b). These considerations are consistent with broader ASD stabilization principles, in which reduced molecular mobility, drug–carrier interactions, and suppression of nucleation and crystal growth are central determinants of physical stability (Baghel et al., 2016; Pandi et al., 2020; Sahoo et al., 2020; Zhang et al., 2023).

Achieving sufficient carrier mobility during irradiation does not guarantee that the amorphous drug will remain molecularly dispersed after cooling or equilibration (Lalge et al., 2023; Lehmkemper et al., 2017b). The cooling period is therefore important, because it determines how rapidly the carrier changes from a mobile state that supports drug dissolution to a less mobile state that can immobilize the amorphous drug (Lalge et al., 2023). The final matrix must limit recrystallization over the relevant interval between microwave treatment and administration. Although this interval is shorter than the shelf-life required for conventional ASDs, it remains critical to preserve the dissolution advantage of the amorphous phase.

Recrystallization has also been observed directly in microwave-generated systems. Celecoxib recrystallized during cooling in PEG-only formulations, whereas incorporation of PVP improved retention of the amorphous state (Hempel et al., 2021d). Similarly, in glycerol-containing indomethacin systems, a formulation containing 20% drug remained amorphous after 2 weeks under ambient conditions, whereas a supersaturated formulation containing 30% drug showed recrystallization over the same period (Hempel et al., 2021e).

Incomplete conversion also has implications for post-amorphization stability. Residual crystalline drug in microwave-treated formulations should not be considered solely as an unconverted fraction that reduces the degree of amorphization. In amorphous solid dispersions, residual crystalline domains may facilitate further crystallization by providing pre-existing surfaces for crystal growth (Moseson et al., 2021; Moseson and Taylor, 2023). When sufficient molecular mobility remains within the surrounding amorphous phase, drug molecules can be incorporated into existing crystals, resulting in crystal growth and progressive loss of the amorphous fraction. This mechanism provides a rationale for evaluating residual crystallinity together with post-treatment stability in microwave-generated systems, although the extent of subsequent crystal growth must be established for each formulation under the intended holding conditions.

Residual crystallinity can also affect dissolution performance. Its impact depends not only on the crystalline fraction but also on the properties of the residual crystals and the ability of the polymer to inhibit their growth (Moseson et al., 2020; Que et al., 2018). In hot-melt-extruded bicalutamide–PVPVA ASDs, residual crystals promoted matrix crystallization during dissolution and reduced supersaturation under non-sink conditions (Moseson et al., 2021). The consequence of crystallinity in a nominally amorphous product has also been demonstrated clinically: following deliberately induced crystallization during storage, a marketed generic tacrolimus product containing 20% or 50% crystalline drug failed to meet bioequivalence criteria for Cmax and AUC relative to the amorphous generic product in healthy participants (Taylor et al., 2025). Although this system was not microwave-generated, it indicates that drug crystallinity in an ASD can affect *in vivo* performance. These findings support assessing the performance consequences of any residual crystallinity in microwave-treated formulations, while distinguishing crystallization during dissolution from recrystallization during post-treatment storage. Consequently, residual crystalline content should be evaluated not only as an indicator of conversion efficiency but also as a potential risk factor for post-treatment recrystallization and impaired dissolution performance. Strategies that minimize residual crystal domains, enhance drug–carrier miscibility, or increase intermolecular interactions capable of suppressing crystal growth may therefore improve the robustness of microwave-induced amorphization.

From a pharmaceutical performance perspective, stabilization of the generated amorphous phase should be evaluated together with the extent of amorphization rather than considered as an independent attribute. Reported microwave-induced systems have demonstrated substantial differences in amorphous conversion and post-treatment behavior depending on drug loading, carrier composition, and activation strategy. Moreover, highly amorphized systems do not necessarily provide improved dosage-form performance when microwave treatment induces excessive matrix softening, fusion, or impaired disintegration (Holm et al., 2022a; Holm et al., 2022b). Therefore, practical evaluation of microwave-induced amorphization should include not only amorphous fraction and recrystallization tendency but also the preservation of tablet structure, disintegration behavior, dissolution performance, and stability over the intended post-activation administration period.

Overall, microwave-induced *in situ* amorphization can be viewed as a sequence of coupled mechanistic events: dielectric activation, carrier plasticization, drug dissolution and diffusion, and post-amorphization stabilization. Failure at any step can limit the final outcome. A formulation that absorbs microwave energy but does not generate sufficient mobility may remain crystalline, whereas a formulation that generates mobility but lacks adequate stabilization may fail to retain the generated amorphous phase. The design objective is to coordinate energy absorption, transient carrier mobility, and subsequent immobilization within the same dosage form. This framework motivates excipient strategies that deliberately combine microwave responsiveness, transient mobility generation, and stabilization capacity, as examined in the following section.

## 4 Drug, excipient, and formulation design strategies

Microwave-induced *in situ* amorphization requires coordination between microwave responsiveness, transient mobility generation, and post-amorphization stabilization within a single formulation (Hempel et al., 2021c; Hempel et al., 2021d; Hempel et al., 2021e; Qiang et al., 2020). A microwave-responsive component may improve energy absorption without providing sufficient drug dissolution or post-amorphization stability, whereas a stabilizing carrier may fail if it does not become sufficiently mobile during irradiation (Hempel et al., 2021c; Hempel et al., 2021d; Hempel et al., 2021e). Excipient selection should therefore reflect the balance among these functions, rather than any single property.

The activation routes summarized in Fig. 5 provide different starting points for formulation design. Sorbed water, glycerol, and non-ionic surfactants can contribute to dielectric coupling and plasticization, whereas crystalline hydrates and thermolytic salts provide internal activation sources (Doreth et al., 2017; Hempel et al., 2021c; Hempel et al., 2021d; Hempel et al., 2021e; Holm et al., 2021; Qiang et al., 2023; Qiang et al., 2024). PEG-based systems provide another non-aqueous route by forming a mobile carrier phase during irradiation (Hempel et al., 2021b; Hempel et al., 2021d). The value of each strategy depends on whether it creates a transient processing window in which the carrier is mobile enough for drug dissolution during irradiation but sufficiently stable after irradiation.

Drug-related properties also determine the feasibility of microwave-induced *in situ* amorphization. Because amorphization is governed by dissolution into a transiently mobile carrier phase, not by direct drug melting, the relevant drug-related variables include drug loading, crystal particle size, drug–carrier miscibility, glass-forming ability, and crystallization tendency (Edinger et al., 2018; Hempel et al., 2020b; Holm et al., 2022a; Lalge et al., 2023). A high drug fraction may exceed the carrier's capacity for dissolution and molecular dispersion, limiting the maximum achievable amorphous fraction. Consistent with this interpretation, celecoxib–PVP tablets showed a marked decrease in relative amorphous fraction, from 90 ± 7% at 10% drug loading to 31 ± 7% at 50% drug loading, after the same microwave treatment (Edinger et al., 2018). The effect of drug loading is therefore formulation-dependent and should be evaluated under the actual activation conditions, rather than inferred solely from ambient-temperature drug–carrier solubility (Holm et al., 2022a). Drugs with poor carrier miscibility or a strong crystallization tendency may also require a larger stabilizing carrier fraction or more effective plasticization during irradiation, consistent with the general principles of ASD stabilization (Baghel et al., 2016; Pandi et al., 2020; Sahoo et al., 2020; Zhang et al., 2023). Drug selection and excipient selection should therefore be treated as coupled formulation decisions, rather than separate screening steps.

Table 3 summarizes the strategies that are often combined within the same formulation. An activating or plasticizing component may be paired with a polymeric carrier that contributes to post-amorphization stabilization (Hempel et al., 2021d). Water can be effective, because it contributes to both microwave coupling and plasticization, but externally conditioned systems remain sensitive to moisture uptake, water distribution, and residual plasticization after irradiation (Hempel et al., 2020a; Hempel et al., 2021c; Lehmkemper et al., 2017a). PEG-based systems can promote amorphization by generating a mobile phase during irradiation, but excessive matrix mobility may increase the risk of post-treatment recrystallization (Hempel et al., 2021d). Glycerol and non-ionic surfactants provide additional examples of this functional overlap, supporting microwave responsiveness, carrier plasticization, and drug solubilization to different extents (Hempel et al., 2021e; Qiang et al., 2023).

**Table 3:** Formulation design requirements for microwave-induced *in situ* amorphization.

Design requirement	Formulation approach	Relevant components	Principal trade-off	Reference
Microwave coupling	Polar-component dielectric coupling	Sorbed water, glycerol, non-ionic surfactants	Moisture dependence, residual plasticization	(Doreth et al., 2017; Hempel et al., 2021c; Hempel et al., 2021e; Qiang et al., 2023)
	Internal activation sources	Crystalline hydrates, thermolytic salts	Release control, residual components	(Holm et al., 2021; Qiang et al., 2024)
Transient carrier mobility	Plasticization, melt formation, viscosity reduction	Water, PEG, glycerol, surfactants	Deformation, residual mobility	(Doreth et al., 2017; Hempel et al., 2021b; Hempel et al., 2021c; Hempel et al., 2021d; Hempel et al., 2021e; Qiang et al., 2023)
Drug dissolution	Increased contact, shorter diffusion distance, improved miscibility	PVP, PEG–PVP matrices, surfactant-containing systems	Drug-loading dependence, poor contact, incomplete conversion	(Edinger et al., 2018; Hempel et al., 2020b; Hempel et al., 2021d; Holm et al., 2022a; Qiang et al., 2021; Qiang et al., 2023)
Amorphous-phase stabilization	Matrix rigidity, drug–carrier interactions	PVP, PEG–PVP, polymer-rich matrices	Rigidity *versus* activation-stage mobility	(Doreth et al., 2017; Doreth et al., 2018; Hempel et al., 2021d)

Within this balance, polymer grade and molecular weight are important, because they influence both the transient mobility generated during irradiation and the rigidity of the matrix after treatment (Doreth et al., 2018). Composite formulations provide one practical route to reconciling these competing demands by distributing activation, mobility, and stabilization functions across complementary excipients within the same dosage form. PEG–PVP systems exemplify this division of labor: PEG forms a mobility-enhancing melt phase, while PVP provides the stabilizing matrix that limits recrystallization (Hempel et al., 2021d).

These drug- and excipient-related principles define the formulation space in which microwave-induced *in situ* amorphization can occur. Whether this potential is realized reproducibly depends on the irradiation and process variables applied to the selected formulation.

## 5 Process parameters and analytical considerations

Reproducible microwave-induced *in situ* amorphization requires irradiation conditions that produce an appropriate thermal, physical, and solid-state response in the intact dosage form. Microwave power, exposure time, moisture content, temperature profile, and compact structure must therefore be evaluated in relation to the formulation. Differences in microwave coupling efficiency, carrier mobility, drug–carrier miscibility, and resistance to deformation limit the applicability of a single fixed set of instrument settings across formulations. The formulation-specific operating window is defined here as the range of irradiation and process conditions within which sufficient amorphous conversion is achieved, without compromising compact integrity, spatial uniformity, or post-treatment quality.

Nominal microwave settings provide only a starting point for process development (Doreth et al., 2017; Hempel et al., 2021b). The same applied settings can produce different thermal responses, depending on the formulation composition, water content, compact size, density, and dielectric responsiveness (Heng et al., 2010; Zhang et al., 2026). Because nominal power and exposure time do not directly represent the energy absorbed by the compact, comparisons across microwave devices and laboratories are difficult unless device configuration, sample positioning, and temperature–time history are adequately reported (Hempel et al., 2021b; Hempel et al., 2021c; Pitchai et al., 2012). Sufficient exposure is required for dielectric activation, carrier softening, and drug dissolution into the carrier phase. The practical balance is to minimize residual crystallinity without inducing deformation, water loss, or non-uniform conversion. Optimization should therefore consider power and exposure time as coupled process variables.

In water-mediated systems, moisture content is simultaneously a formulation attribute and a process variable. Sorbed water can support dielectric activation during irradiation (Doreth et al., 2017; Hempel et al., 2021c). In addition, water-induced plasticization can lower the glass-transition temperature and increase polymer mobility (Hancock and Zografi, 1994). In such systems, humidity conditioning, water uptake, water distribution, and moisture loss during irradiation can all affect the outcome. Water content must be controlled to maintain microwave responsiveness and carrier mobility, without increasing deformation or spatial heterogeneity. This requirement is particularly relevant to PVP-based systems, where water-dependent plasticization can promote amorphization, but also increase sensitivity to humidity conditioning and residual mobility. Convective-heating experiments further illustrate the importance of water-mediated mobility: conditioned celecoxib–PVP compacts containing approximately 22% water reached 92.2 ± 0.2% amorphization after 20 min at 100 °C, whereas dry compacts containing approximately 4% water reached only 14.3 ± 1.4% amorphization under the same thermal treatment (Hempel et al., 2020a). However, such high water content may not be directly compatible with conventional tablet handling, mechanical robustness, or chemical stability requirements, helping explain why internally activated systems have been explored as alternatives to external humidity conditioning.

Temperature measurements provide a useful process readout only when interpreted in relation to the carrier state. A measured temperature should not be treated as direct evidence of amorphization; its relevance depends on whether it corresponds to a carrier state that can dissolve the drug (Hempel et al., 2020a). Bulk temperature measurements may not capture local heating, water redistribution, or spatial differences in carrier mobility within the compact. A temperature–time profile is therefore more informative than a single endpoint measurement or maximum temperature.

The required processing window also depends on the selected drug–carrier system. Although amorphization is carrier-mediated, drug melting behavior may still influence the practical temperature–time window required for conversion. This possibility has not yet been directly examined in the available literature, as the model drugs studied to date fall within a relatively narrow melting-point range. Polymer grade, molecular weight, and viscosity also influence how a selected formulation responds to irradiation by affecting carrier mobility (Doreth et al., 2018; Hempel et al., 2021b). For example, moisture-conditioned PVP K12, K17, and K25 showed glass transition temperatures (*T*_*g*_) of 16.4 ± 2.2 °C, 39.6 ± 0.8 °C, and 50.3 ± 2.1 °C, respectively, while substantial *in situ* amorphization was observed only in the PVP K12 compact under the tested conditions (Doreth et al., 2018). Because the treatment temperature exceeded the *T*_*g*_ of all three grades, the position of the treatment temperature relative to *T*_*g*_ alone does not account for these differences. Carrier viscosity and molecular mobility above *T*_*g*_, both of which are influenced by polymer molecular weight, may therefore be more directly related to the rate at which the drug dissolves into the carrier. Formulation composition and irradiation conditions should therefore be optimized together, rather than independently.

Compact structure is process-relevant, because microwave-induced *in situ* amorphization occurs in the final or near-final compact. Tablet size, weight, and thickness can influence microwave heating, heat dissipation, and the spatial uniformity of amorphization, indicating that these characteristics should be considered process-relevant formulation variables, rather than passive dosage-form attributes (Zhang et al., 2026). Although the effects of compact density, porosity, and internal drug–carrier distribution have not yet been systematically quantified in microwave-induced *in situ* amorphization, these characteristics are mechanistically plausible process variables. Material density can influence the dielectric properties and microwave-heating behavior of pharmaceutical materials (Heng et al., 2010), while drug–polymer contact and particle size can affect dissolution-driven microwave-induced amorphization (Hempel et al., 2020b). These structural variables could also plausibly influence cooling behavior after irradiation, although this has not yet been directly quantified in microwave-induced *in situ* amorphization. Excessive deformation, cracking, particle fusion, or heterogeneous conversion can compromise both process interpretation and dosage-form acceptability. The importance of preserving dosage-form architecture is illustrated by the multiparticulate systems shown in Fig. 7. Although the overall tablet geometry was largely retained after microwave treatment, X-ray micro-computed tomography revealed substantial changes in internal structure as microwave power increased (Holm et al., 2022b). At 800 W, individual drug–polymer granules remained comparatively distinguishable, whereas treatment at 1000 W produced greater granule fusion, structural rearrangement, fracture formation, and low-density regions, particularly in MCC-containing tablets. These findings demonstrate that apparent preservation of external tablet geometry does not necessarily indicate preservation of the internal matrix structure and help explain why extensive amorphization may be accompanied by altered mechanical properties, impaired disintegration, and modified drug release. In the reported systems, processing conditions that produced extensive or complete amorphization were also associated with fusion, impaired disintegration, and delayed drug release (Holm et al., 2022a; Holm et al., 2022b). Because the solid-state characterization of milled powders may obscure changes in dosage-form structure and the associated barriers to disintegration, process evaluation should include intact compacts.

**Fig. 7:**
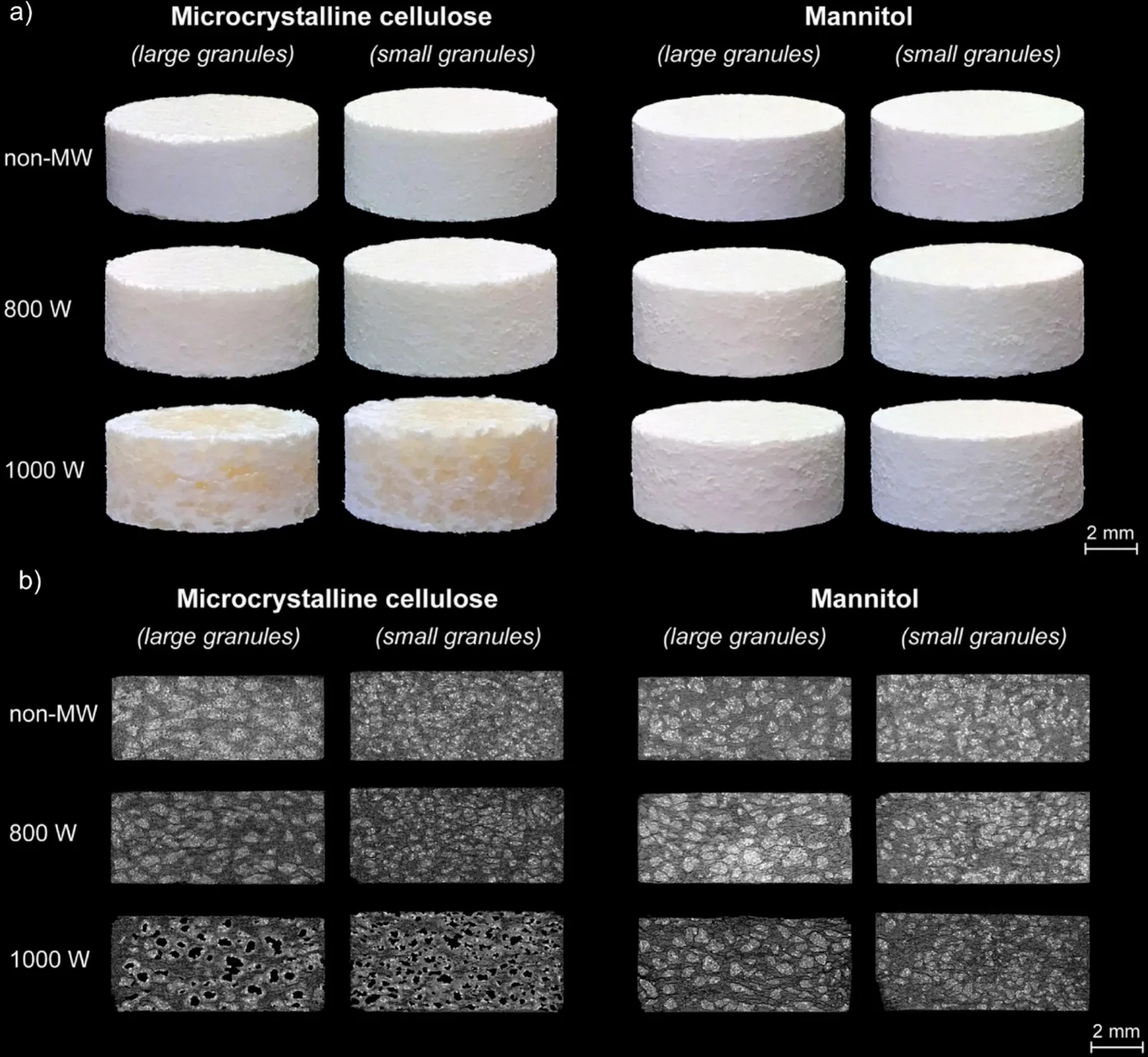
Effects of formulation architecture and microwave irradiation on multiparticulate tablet structure. (a) Representative photographs of tablets containing microcrystalline cellulose (MCC) or mannitol (MAN) as the extragranular filler and large or small drug–polymer granules, before treatment (non-MW) and after irradiation at 800 or 1000 W for 10 min. Overall tablet geometry was retained after treatment. (b) Corresponding reconstructed X-ray micro-computed tomography (XμCT) cross-sections. Granules remained largely distinguishable at 800 W, whereas treatment at 1000 W produced marked granule fusion and low-density pockets in MCC-based tablets. In MAN-based tablets, granules remained separated, although fractures were observed. MCC tablets containing large and small granules were compressed at 200 and 100 MPa, respectively; corresponding MAN tablets were compressed at 250 and 150 MPa. Lighter and darker regions in XμCT images represent relatively higher and lower density, respectively. Adapted from Holm et al. (2022b), licensed under CC BY 4.0.

Analytical evaluation should first establish the extent of solid-state conversion after microwave treatment. While XRPD and DSC can detect changes in crystallinity, they may have limited sensitivity to low levels of residual crystallinity or local heterogeneity (Chasse et al., 2022; Dedroog et al., 2020; Moseson et al., 2021; Moseson and Taylor, 2023). Dissolution testing has also been used to detect crystallinity in amorphous solid dispersions, but its discriminating ability depends on the crystallization tendency of the drug, drug loading, polymer, and sink conditions (Purohit et al., 2023). It is therefore best regarded as a complement to, rather than a replacement for, solid-state analysis. Transmission Raman spectroscopy combined with partial least squares modeling has also been used to quantify the degree of microwave-induced amorphization in intact tablets (Edinger et al., 2018). Such non-destructive approaches may complement conventional solid-state analyses, especially when sample preparation or destructive analysis could affect the interpretation of conversion.

Post-treatment testing extends process evaluation beyond immediate solid-state conversion. The amorphous phase generated during irradiation must remain sufficiently stable over the intended interval before administration, as discussed in Section 3.4. Chemical stability should also be evaluated, because microwave exposure and moisture-containing systems may affect drug content or related-substance profiles (Qiang et al., 2021; Qiang et al., 2024). Accordingly, post-treatment evaluation should integrate solid-state quantification with assessment of dosage-form integrity, disintegration and dissolution, and physical and chemical stability (Holm et al., 2022a; Holm et al., 2022b; Zhang et al., 2021). Fig. 8 outlines a sequential process and analytical evaluation framework for microwave-induced *in situ* amorphization, while Table 4 summarizes the corresponding key variables, readouts, and evaluation focus.

**Fig. 8:**
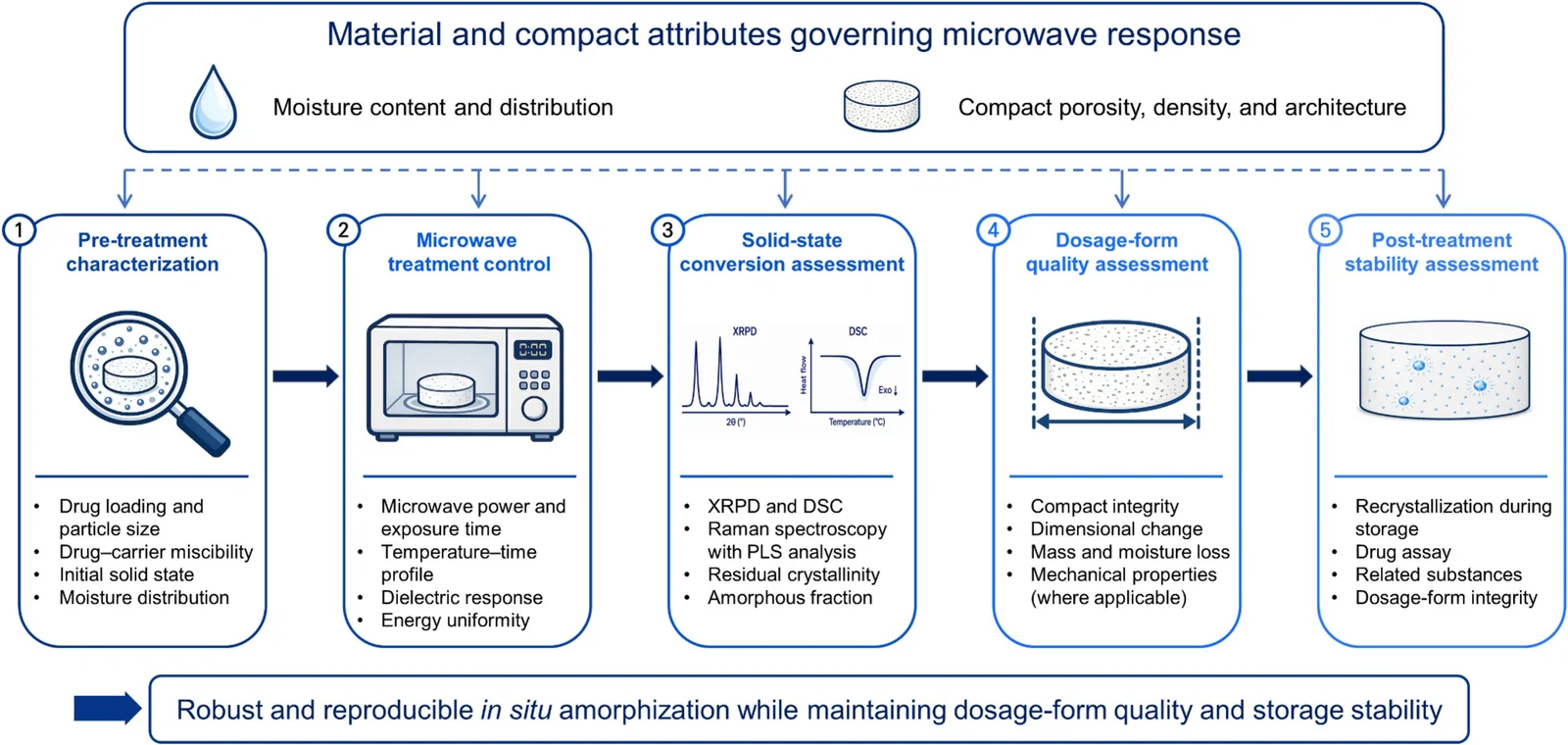
Process and analytical evaluation framework for microwave-induced *in situ* amorphization. The framework links pre-treatment characterization, microwave treatment control, solid-state conversion assessment, dosage-form quality assessment, and post-treatment stability assessment. Moisture content/distribution and compact structure are shown as cross-cutting formulation attributes.

**Table 4:** Key process and analytical variables for microwave-induced *in situ* amorphization.

Parameter or readout	Process relevance	Control consideration	Reference
Microwave power	Energy input rate	Overheating, incomplete conversion	(Doreth et al., 2017; Holm et al., 2022b)
Exposure time	Irradiation duration	Deformation, water loss, non-uniform conversion	(Doreth et al., 2017; Hempel et al., 2020a; Hempel et al., 2021c)
Moisture content	Dielectric coupling, plasticization	Insufficient responsiveness, residual mobility	(Doreth et al., 2017; Hempel et al., 2021c)
Moisture distribution	Heating uniformity	Local under- or over-processing	(Doreth et al., 2017; Hempel et al., 2021c)
Temperature–time profile	Carrier-state evolution	Temperature trajectory, endpoint limitation	(Hempel et al., 2021b; Hempel et al., 2020a; Hempel et al., 2021c)
Compact structure	Heating and deformation behavior	Size, weight, thickness, internal architecture; density and porosity as plausible variables	(Holm et al., 2022b; Zhang et al., 2021; Zhang et al., 2026)
Crystallinity confirmation	Amorphous conversion	Residual crystallinity	(Doreth et al., 2017; Doreth et al., 2018; Hempel et al., 2021d; Hempel et al., 2021e; Qiang et al., 2021; Qiang et al., 2024)
Intact-tablet assessment	Non-destructive conversion assessment	Residual crystalline regions	(Edinger et al., 2018)
Dissolution performance	Drug-release behavior	Disintegration time, drug-release profile	(Doreth et al., 2017; Holm et al., 2022a; Holm et al., 2022b; Zhang et al., 2026)
Physical stability	Amorphous retention	Short-term recrystallization	(Hempel et al., 2021d; Hempel et al., 2021e)
Chemical stability	Degradation susceptibility	Assay loss, related substances	(Qiang et al., 2021; Qiang et al., 2024)

Microwave treatment can modify tablet performance through matrix softening, polymer fusion, component redistribution, and changes in porosity (Holm et al., 2022b). Therefore, amorphous conversion alone cannot be considered a sufficient indicator of dosage-form performance; successful processing requires a balance between amorphization and preservation of mechanical integrity, disintegration, and drug-release properties.

The impact of microwave irradiation on tablet mechanical properties is formulation-dependent. Zhang et al. (2021) reported higher hardness for microwave-generated amorphous indomethacin tablets containing Soluplus® and HPC HF than for the corresponding directly compressed tablets, whereas formulations containing HPMC, HPMCAS, Eudragit® E PO, and PEO showed lower hardness. Thus, microwave treatment does not universally strengthen or weaken tablets, and the outcome depends on carrier properties and matrix behavior during irradiation.

In multiparticulate systems, extensive amorphization may also alter dosage-form functionality (Holm et al., 2022b). Holm et al. (2022b) reported tensile-strength ranges of 0.7–1.7 MPa before treatment and 0.4–3.7 MPa after treatment across different conditions. MCC-containing tablets increased in strength after 1000 W treatment, whereas mannitol-containing tablets weakened. However, complete amorphization did not necessarily improve dosage-form performance. MCC tablets containing large granules and compacted at 100 MPa disintegrated in 0.2 min before treatment but did not completely disintegrate within 30 min after irradiation at 1000 W for 10 min, indicating that structural changes during activation may hinder drug release.

Microwave-induced changes in tablet structure also influence dissolution behavior. Holm et al. (2022b) showed that microwave-treated MCC formulations achieved higher maximum drug concentrations than untreated crystalline tablets, but matrix fusion and impaired disintegration affected release performance. Similarly, fully amorphous high-loading celecoxib systems exhibited slower initial release than physical mixtures, with supersaturation developing only during prolonged dissolution testing (Holm et al., 2022a). In contrast, partial amorphization of indomethacin–PVP tablets produced an intrinsic dissolution rate comparable to a fully amorphous glass solution (Doreth et al., 2017).

These differences highlight that dissolution outcomes depend on the evaluation method (Hermans et al., 2022; Li and Taylor, 2018). Intrinsic dissolution reflects release from a controlled surface area, whereas intact tablet dissolution additionally depends on wetting, swelling, erosion, disintegration, and diffusion. Likewise, supersaturation measurements represent drug concentration relative to a defined solubility reference and should not be directly interpreted as dosage-form performance.

Therefore, evaluation of microwave-induced *in situ* amorphization should integrate solid-state characterization with mechanical testing, disintegration assessment, and dissolution profiling under clearly defined conditions. Parameters including dissolution medium, pH, volume, agitation, sink conditions, sampling duration, and tested dosage-form state should be reported. Increased amorphous fraction or supersaturation alone should not be considered sufficient evidence of improved pharmaceutical performance.

These variables should not be optimized in isolation. Moisture content, temperature history, drug–carrier properties, and compact geometry can alter the effect of a given power–time setting on carrier mobility, amorphous conversion, and dosage-form integrity. Establishing formulation-specific conditions on this basis is therefore essential for moving microwave-induced *in situ* amorphization beyond proof-of-concept studies toward reproducible dosage-form preparation.

## 6 Future perspectives

Microwave-induced *in situ* amorphization has progressed from an initial proof-of-concept toward a formulation-dependent strategy for generating amorphous dispersions within compacted dosage forms. Further progress requires moving from system-specific demonstrations to reproducible design principles.

### 6.1 Defining the applicable drug–excipient space

The applicable drug–excipient space remains insufficiently defined. Although multiple polymer matrices and formulation strategies have been explored, most studies have focused on a limited set of model drugs. Broader evaluation of drugs with different melting behavior, glass-forming ability, crystallization tendency, molecular size, polarity, and carrier miscibility will be needed to determine which combinations are most suitable for microwave-induced *in situ* amorphization. Such studies would clarify whether the approach is broadly generalizable across poorly soluble drugs or more appropriate for selected compounds and carrier systems.

### 6.2 Process control and analytical standardization

Formulation-specific operating windows should be systematically mapped across multiple drug–carrier systems, rather than established in isolated case studies. Such studies would allow processing requirements to be compared between formulations and may help identify variables that consistently govern successful conversion.

Analytical development should focus less on introducing additional characterization tools and more on standardizing how conversion is assessed and reported. Standardized reporting criteria for residual crystallinity, amorphous fraction, dosage-form integrity, and post-treatment stability would make results easier to compare across formulations, devices, and laboratories.

Beyond standardizing microwave-based studies, comparisons with related triggered *in situ* amorphization approaches, including laser-assisted and magnetic hyperthermia systems, could broaden the mechanistic interpretation of microwave-induced systems. Because these approaches rely on different energy transducers but share similar formulation-level challenges, cross-platform comparison may help distinguish general requirements, such as carrier mobility and drug–carrier miscibility, from trigger-specific constraints (Ansari et al., 2022; Hempel et al., 2021a).

### 6.3 Dosage form design for flexible dosing

Compact size and configuration become particularly important when microwave-induced *in situ* amorphization is considered for flexible dosing. Recent mini-tablet studies suggest that these factors may be especially relevant for pediatric applications (Zhang et al., 2026).

In this context, microwave-induced *in situ* amorphization should be distinguished from three-dimensional printing, which has been explored for dose tailoring, geometry control, and the amorphous formulation of poorly soluble drugs during dosage-form fabrication (Kim et al., 2021; Kreft et al., 2024). Its potential role would instead be to enable late-stage amorphization of preformed units whose number, size, or configuration may be adjusted according to dosing needs. The technology should not yet be regarded as an established personalized medicine platform, but it may be compatible with dose-flexible or small-batch preparation, provided that reproducible activation, dosage-form quality, and post-treatment stability can be demonstrated.

### 6.4 Translational requirements and pharmaceutical relevance

In the initial proof-of-concept study, microwave-treated indomethacin–PVP compacts showed a 6.3-fold higher intrinsic dissolution rate than the corresponding physical mixture, indicating that solid-state conversion may translate into improved dissolution behavior (Doreth et al., 2017). However, later studies showed that high amorphous conversion does not necessarily translate into rapid drug release when microwave treatment produces fused or poorly disintegrating matrices (Holm et al., 2022a; Holm et al., 2022b). These findings indicate that amorphous conversion alone is not an adequate predictor of dosage-form performance and should be evaluated together with disintegration and dissolution behavior. Future studies should therefore determine whether formulations optimized for both amorphous conversion and release performance can improve *in vivo* bioavailability.

Beyond *in vivo* evaluation, translational development will require characterization of the activation procedure, and definition of a robust operating window. Nominal power settings may not directly reflect the energy absorbed by the dosage form, because heating behavior can be influenced by electromagnetic field distribution within the cavity, sample placement or movement, load conditions, and power-delivery mode (Yan et al., 2025). Calibrated microwave systems may be useful during development to relate device settings to dosage-form response and establish reference conditions, but they need not represent the device ultimately used by patients. The translational objective is instead to design formulations and dosage forms that achieve reliable conversion across a representative range of heating conditions expected during use.

The location of activation is a key translational consideration. Activation at the manufacturing site would largely forfeit the advantage of maintaining the drug in a crystalline state during storage and distribution, whereas point-of-care activation may be appropriate when supervised handling is required. For products intended to be amorphized immediately before administration, household microwave activation represents a potential point-of-use scenario, because it would allow the drug to remain crystalline until the point of use without requiring supervised handling (Doreth et al., 2017). However, translation to patient-operated activation introduces additional risks associated with variability in microwave exposure. Under-treatment, caused by insufficient heating time, inappropriate power selection, or poor positioning within the microwave cavity, may result in incomplete amorphization and persistence of crystalline drug domains (Doreth et al., 2017; Edinger et al., 2018). These residual crystals can diminish the solubility advantage associated with the amorphous state and, depending on the drug–polymer system and extent of polymer-mediated crystallization inhibition, may serve as sites for subsequent crystal growth (Moseson et al., 2020). Conversely, excessive microwave exposure may subject the dosage form to unnecessary thermal stress, raising concerns regarding chemical degradation in thermolabile compounds, moisture loss, and physical changes such as excipient melting, densification, or matrix deformation (Davis et al., 2021; Hempel et al., 2021c; Hempel et al., 2021d).

A further challenge is ensuring uniform solid-state conversion throughout the dosage form. Although API-content uniformity must be established for the precursor compact, activation-related variability in solid-state conversion and drug release must also be considered within and between dosage units. If uneven or excessive heating causes degradation, the amount of intact drug per unit may also vary. Development studies should therefore assess post-activation assay, degradation products, and dosage-unit uniformity where relevant (ICH, 1999). Domestic microwave ovens exhibit spatially non-uniform electromagnetic fields, and heating behavior is influenced by sample position, rated power, and cavity dimensions (Pitchai et al., 2012). Because microwave-induced amorphization is strongly temperature dependent and requires attainment of a sufficient temperature threshold (Hempel et al., 2021c; Hempel et al., 2021d), such heating heterogeneity could potentially produce spatial variation in amorphization within a dosage unit.

Translation will therefore depend on developing formulations and dosage forms that provide a sufficiently broad activation window to accommodate realistic differences among household microwave ovens while still permitting simple instructions for use based on validated combinations of nominal power and exposure time. This will require systematic testing across representative devices and operating conditions, confirmation of reproducible conversion, and safeguards against insufficient or excessive microwave treatment. From a regulatory perspective, patient-operated microwave activation would introduce an additional product-preparation step that would need to be considered within the pharmaceutical development, control strategy, stability assessment, and instructions for use. Regulatory evaluation would therefore be expected to address the reproducibility and robustness of the activation procedure, chemical integrity following treatment, and risks associated with variability in patient operation (ICH, 2009; ICH, 2023). Where the extent of amorphization or residual crystallinity is demonstrated to affect dissolution, stability, bioavailability, or other aspects of product performance, these solid-state characteristics could constitute critical quality attributes or otherwise require appropriate control through direct or surrogate specifications (ICH, 1999). Assay, degradation products, and relevant post-activation stability attributes would likewise require appropriate characterization (ICH, 2003; ICH, 1999). Thus, household microwave activation should be regarded not merely as a convenience step, but as a controlled point-of-use product transformation whose operating conditions and resulting product quality would need to be sufficiently robust and reproducible for the intended patient-use setting.

Because microwave-induced *in situ* amorphization shifts part of the solid-state transformation closer to the point of use, the process must ultimately be evaluated not only as a formulation strategy but also as a controlled dosage-form preparation procedure. The allowable interval between activation and administration, together with post-activation physical and chemical stability, will be an important part of this evaluation. Meeting these requirements will determine whether the approach can become a useful complement to conventional ASD manufacturing, particularly where long-term amorphous-state storage remains a major challenge.

## 7 Conclusion

Microwave-induced *in situ* amorphization represents a distinct delayed-amorphization strategy for improving the oral delivery of poorly water-soluble drugs by postponing the solid-state transformation until after preparation of the final dosage form, thereby bringing it closer to the point of use. The reviewed studies indicate that successful conversion depends on coordinating dielectric activation, carrier mobility, and post-amorphization stabilization, with the balance among these processes determined by the drug, excipient system, and processing conditions. Although the field has advanced beyond initial proof-of-concept studies, broader evaluation across diverse drugs and formulation systems and greater standardization of analytical and performance criteria are still needed. Translation will also require characterization of device-dependent activation, demonstration of robust operating windows specific to the formulation and dosage form, and confirmation of *in vivo* bioavailability. Microwave-induced *in situ* amorphization may therefore complement rather than replace conventional ASD manufacturing for selected poorly soluble drugs, particularly where long-term amorphous-state storage or downstream processing of metastable systems remains challenging.

## CRediT authorship contribution statement

**Kiran Suwal:** Writing – original draft, Investigation, Data curation. **Du Hyung Choi:** Writing – original draft, Visualization, Investigation. **Ji Min Choi:** Investigation. **Seong Hoon Jeong:** Methodology, Investigation. **Nam Ah Kim:** Writing – review & editing, Conceptualization. **Ki Hyun Kim:** Writing – review & editing, Supervision, Funding acquisition, Conceptualization.

## Declaration of competing interest

The authors declare that they have no known competing financial interests or personal relationships that could have appeared to influence the work reported in this paper.

## Data Availability

No data was used for the research described in the article.
